# Quantitative Analysis of Performance Recovery in Semi-Professional Football Players after the COVID-19 Forced Rest Period

**DOI:** 10.3390/s22010242

**Published:** 2021-12-29

**Authors:** Luigi Truppa, Lorenzo Nuti, Stefano Mazzoleni, Pietro Garofalo, Andrea Mannini

**Affiliations:** 1 The BioRobotics Institute, Scuola Superiore Sant’Anna, 56127 Pisa, Italy; 2 Department of Excellence in Robotics & AI, Scuola Superiore Sant’Anna, 56127 Pisa, Italy; 3 IRCCS Fondazione don Carlo Gnocchi, 50143 Firenze, Italy; amannini@dongnocchi.it; 4 School of Biomedical Engineering, Università di Pisa, 56127 Pisa, Italy; lornuti18@gmail.com; 5 Department of Electrical Engineering and Information Technology, Politecnico di Bari, 70125 Bari, Italy; stefano.mazzoleni@poliba.it; 6 Turingsense EU Lab s.r.l., 47121 Forli, Italy; pietro.garofalo@turingsense.com

**Keywords:** wearable sensors, performance recovery, explosive and ballistic assessment, sport

## Abstract

This study proposes the instrumental analysis of the physiological and biomechanical adaptation of football players to a fatigue protocol during the month immediately after the COVID-19 lockdown, to get insights into fitness recovery. Eight male semi-professional football players took part in the study and filled a questionnaire about their activity during the lockdown. At the resumption of activities, the mean heart rate and covered distances during fatiguing exercises, the normalized variations of mean and maximum exerted power in the Wingate test and the Bosco test outcomes (i.e., maximum height, mean exerted power, relative strength index, leg stiffness, contact time, and flight time) were measured for one month. Questionnaires confirmed a light-intensity self-administered physical activity. A significant effect of fatigue (Wilcoxon signed-rank test *p* < 0.05) on measured variables was confirmed for the four weeks. The analysis of the normalized variations of the aforementioned parameters allowed the distinguishing of two behaviors: downfall in the first two weeks, and recovery in the last two weeks. Instrumental results suggest a physiological and ballistic (i.e., Bosco test outcomes) recovery after four weeks. As concerns the explosive skills, the observational data are insufficient to show complete recovery.

## 1. Introduction

The COVID-19 pandemic lockdown has definitely changed the world of sports and football in particular. All agonistic (i.e., major and minor football leagues, the Olympic games, and UEFA European Football Championship) and amateur tournaments were canceled or postponed by several nations. Because of strict safety measures adopted by football clubs, athletes in Italy underwent a two-month forced rest period without any form of agonistic training, which is an extremely peculiar situation from a both physical and psychological point of view. Although individual training programs were assigned to football players, the absence of an athletic trainer and the prolonged self-isolation could have played a decisive negative role in the athletes’ physical preparation, especially at the semi-professional level. In general, elite players’ off-season rest period lasts from two weeks to a maximum of four weeks, in which training sessions are still present but reduced in terms of intensity [1,2]. Indeed, it is well established that the reduction in physical activity leads to the loss of muscle mass and a consequent increase in fat mass [3,4,5,6]. In this scenario, it is reasonable to state that the balance of the aforementioned phenomenon moved to atrophy during the lockdown period due to physical inactivity or ineffective training, causing a potential decrease in the performance [1,6].

According to a survey carried out by Pillay et al. on several athletes, 65% of male participants trained every day during COVID lockdown while 23% every alternate day. Nevertheless, the training load and intensity were less intense for the great majority of the athletes [7]. Negative effects of lockdown may even increase in athletes from club sports, in which the training has significant “*social*” components. Indeed, psychological and motivational factors assume an essential role in keeping a healthy fitness and achieving optimal performances [7]. Social isolation, exercise reduction, sedentary behavior, and changes in nutrition also have psychological consequences that can impact sleep and fatigue perception [7]. In addition, it should be stressed, the necessity of a gradual athletes’ re-introduction to the standard training process, aiming at the reduction in injury risk [7,8]. The aim of this work is to instrumentally analyze the physiological and biomechanical adaptation of eight semi-professional football players to a specific fatiguing protocol during the month immediately after the COVID-19 forced rest period, to get insights about the fitness recovery. Starting from the reasonable assumption that the isolation condition had temporarily compromised the muscular tone of the participants [9], the presented article purposes of monitoring the athletes’ performance during the training resumption period and, then, quantify their improvements and the time required to achieve a plateau in the physical strength.

Our opinion is that controlled monitoring of athletes’ response to a constant fatigue protocol could give useful information on their recovery process and, so, the correct timing for a return to sport. In order to achieve this objective, the Wingate test [10], the Bosco test [11], together with the mean heart rate, and Global Navigation Satellite System (GNSS) data measured during the fatigue protocol as a physiological marker were used [12].

Among all the aforementioned parameters, the mean heart rate could give useful information about the physiological adaptation of the participants’ bodies during the duration of the study while the Wingate and Bosco tests allow us to study the evolution of the explosive and ballistic abilities of the athletes and, so, their level of performance. In particular, the Wingate test is an anaerobic test that could be commonly used to quantify anaerobic power for the evaluation of anaerobic fitness levels and to assess anaerobic training effects [13]. It consists of continuous cycling against a preset resistance for a total duration of 30 s. The performance in the Wingate test could be evaluated using an ergometer that gives as output the mean and maximum exerted power. In this scenario, the Wingate test results are extremely useful in assessing the athlete’s explosive capacity. On the other hand, ballistic performance could be defined as the ability to move a certain mass (e.g., athlete’s body) with an impulsive force in a limited amount of time [14], and it assumes a crucial role in a football match, which is characterized by high-intensity intermittent exercises [15]. The most common tests for explosive skills assessment are those that involve vertical jump (i.e., the so-called force tests) [16]. The Bosco test turns out to be one of the most used procedures to quantify such aspects. In particular, the Bosco test is considered a test of anaerobic metabolism and it consists of repeated jumps from a surface [11]. It largely involves the stretch-shortening cycle (SSC) of the lower limb for a relatively long duration (e.g., thirty seconds) [11], and its results are extremely suitable for sports that are characterized by frequent SSC in jumping motions, such as football [17].

This study takes place as a unicum for the instrumental assessment of athletes while regaining physical fitness after a forced stop, given the extraordinary nature of the situation. Indeed, the forced rest period we experienced last year was a mere consequence of the pandemic and it cannot be considered as an extension of the traditional off-season period. In particular, the lockdown happened from March to May, when the competitive activities were still ongoing (while the off-season period is at the end of the activities by definition) and it introduced several additional factors that are not involved in a normal off-season period (i.e., psychological ones). Several studies have already focused on the effects of the pandemic lockdown on athletes, giving useful information on the preventive procedures to carry out for their correct re-introduction in training [18,19]. However, up to our knowledge, only one study has analyzed the effects of the lockdown experienced on athletes [20]. In particular, Grazioli et al. [20] focused on the comparison between the athletes’ performances before, and immediately after, the forced rest period in Brazil, finding that the lockdown was more detrimental than the traditional off-season. Nonetheless, the aim of the present work is quite different from the aforementioned study. As previously indicated, our purpose is to monitor the atheltes’ physiological and explosive abilties in the month immediately after the lockdown to better understand the time required for performance recovery. Despite a multitude of articles analyzing performance after the standard off-season (2–4 weeks), and at the end of the pre-season periods (5–7 weeks) [6,9,21,22], no author has yet monitored athletes’ performance recovery after a long period of physical and physiological difficulties like the pandemic one. We therefore propose a first instrumental analysis of this kind.

## 2. Materials and Methods

### 2.1. Participants

Eight semi-professional football players (USD Grassina, Firenze, Italy. Age: 24.0 ± 3.3 years, height: 180.8 ± 6.3 cm, weight: 72.4 ± 8.3 kg, years of sports practice: 18.0 ± 3.3 years) were involved in tests. Athletes, in accordance with the Helsinki protocol, signed an informed consent form and filled out a questionnaire to take part in the tests. No participant had a record of cardiovascular/respiratory disease nor evidence of neuromuscular diseases. The present study was approved by the joint ethical committee of the Scuola Normale Superiore and Scuola Superiore Sant’Anna (Approval no. 4/2020). Participants filled a questionnaire on their training activities during the forced rest period, to report the type and amount of their physical activities during the 2-month-long lockdown. The short questionnaire also included information about the change in habits in the self-administered training session before and after the lockdown.

### 2.2. Experimental Procedure and Measurements

**Figure 1:**
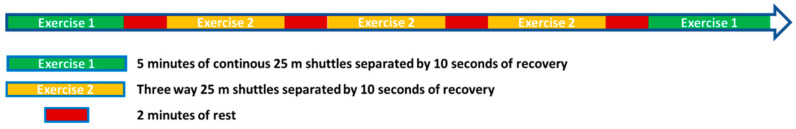
Graphical explanation of the fatigue protocol.

The acquisition protocol was carried out in an uncontrolled environment (e.g., USD Grassina football field, Firenze, Italy). Athletes performed an initial 15 min warm-up phase of dynamic stretching, joint mobility exercises, and core potentiation. The training program in the month of recovery consisted of a fatigue protocol based on high-intensity lactic-acid exercises (shuttles with direction changes) in an uncontrolled environment (e.g., football field) to better simulate standard football training. In particular, the protocol was structured as follows: 5 main phases spaced out by 2 min rest period. In the first and last phases (5 min) athletes had to continuously perform two-way 25 m shuttles in 10 s separated by 10 s recovery time each. The remaining phases are composed of three-way 25 m shuttles to be completed in 10 s, separated by a 10 s recovery time each (Figure 1).

During the training sessions, participants wore a smartwatch (Apple Watch, Apple, Cupertino, CA, USA) for real-time heart rate monitoring and GNSS tracking. In particular, the Apple Watch measures the heart rate by photoplethysmography, which returns an estimation every 5 s [23]. Unfortunately, due to the impossibility of changing the settings on the corresponding app on the smartphone, the sample time remained fixed to 5 s. The Apple watch measured the heart rate continuously during the workout and the returned value (i.e., the mean heart rate during the exercise) was estimated by calculating the average of the 5 s values across all the duration of the workout. The Wingate test was carried out before and after the fatigue protocol: athletes cycled on a mountain bike (Cannondale, Wilton, CT, USA) mounted on an ergometer (Turbo Muin Thru Axle, Elite, Italy) for 30 s at their maximum speed. The Wingate test allowed us to measure the mean and maximum power exerted by the athletes. The pre-fatigue acquisitions were used as a baseline for the assessment of post-fatigue measurements. In addition, a single inertial sensor was placed on the right foot to extract six kinematic parameters from the Bosco test. These parameters were: maximum height, mean exerted power, relative strength index, leg stiffness, contact time, and flight time. The flight time (*T_f_*) and contact time (*T_c_*) during jumps in the Bosco test were directly estimated from the accelerometers of the corresponding Magneto-Inertial Measurement Units (MIMU), as introduced in our previous work [24]. *T_f_* and *T_c_* represent the starting point from which all the other parameters were estimated.

From MIMU signals four groups of parameters were computed:

(i) Time parameters: flight time (*T_f_*) and contact time (*T_c_*). As mentioned before, these two variables represent the starting point from which all the other parameters were calculated;
(ii) Power parameters: mean exerted power;
  (1) $$\begin{array}{c} mean\\ power \end{array}=\frac{mg^{2}}{T_{c}}\left(\frac{T_{f}^{2}}{4}+\frac{T_{c}\left|T_{c}+T_{f}\right|}{\pi }-\frac{T_{c}^{2}}{4}\right)$$
  where *m* is the body mass and $g=9.81\;\left[\frac{m}{s^{2}}\right]$ is gravity acceleration [25].
(iii) Displacement parameters: maximum height (*h_max_* [25]) and reactive strength index (*RSI*) [26];
  (2) $$h_{max}=\frac{g}{8}T_{f}^{2}$$
  (3) $$RSI=\frac{h_{max}}{T_{c}}$$
(iv) Kinematic parameters: leg stiffness (*sfn*), which is the average stiffness of the musculoskeletal system, modeled as a spring, during the ground contact phase [25,27].
  (4) $$stiffness=\frac{m\pi \left(T_{f}+T_{c}\right)}{T_{c}^{2}\left(\frac{T_{f}+T_{c}}{\pi }-\frac{T_{c}}{4}\right)}$$

As for the Wingate test, the aforementioned parameters were estimated before and after the fatiguing protocol to assess their variations due to fatigue. In particular, all the parameters (e.g., heart rate, mean and maximum exerted power, and Bosco test outputs) were measured in training sessions for one month. A preliminary training session (25 May 2020) was carried out to better re-introduce the athletes’ to intense physical activity. This session consisted of a simplified version of the fatigue protocol and was not included in the analysis. The subsequent training sessions were weekly grouped, as follows: two acquisitions during the first (26–28 May 2020) and second weeks (1–3 June 2020) and one acquisition during the third (9 June 2020) and fourth weeks (17 June 2020). In order to define a single set of parameters for each week, the mean values of heart rate, mean and maximum power, and Bosco test outputs were considered in the weeks with more than one acquisition.

### 2.3. Statistical Analysis

An in-depth analysis of the training habits’ effects on performance and physiological adaptation was carried out analyzing the questionnaire information, by reporting descriptive statistics.

The Kolmogorov–Smirnov test was used to test the normality of the distribution of the instrumental parameters. Once it was confirmed that all the parameters belonged to non-gaussian populations, their comparison was carried out using non-parametric tests. In particular, the Wilcoxon signed rank-sum test was used to verify if there was a difference between pre and post-effort parameters each week. Furthermore, the effects of fatigue on the Wingate performance were estimated using the Cliff’s Delta effect size.

In addition, the temporal evolution of the parameters was evaluated through Friedman’s test, which corresponds to repeated measures non-parametric one-way ANOVA, and the Bonferroni test for the post hoc analysis. This statistical test allowed the assessment of whether the athletes underwent a recovery in physical strength by choosing the variable “time” as the test comparison way. The same comparison was carried out on GNNS data about the total distance covered each day, in order to evaluate the reproducibility of the fatigue protocol. To assess the inter-subject variability, the Coefficients of Quartile Variations (CQV) of the mean heart rate and total covered distance were computed. As concerns power parameters and Bosco test outputs, their normalized variations were considered in this study to reduce the effect of inter-subject variability.

The aforementioned tests were carried out with the software “Matlab” (The Mathworks Inc., Natick, MA, USA) assuming a significance threshold α = 0.05.

## 3. Results

### 3.1. Questionnaire

The results of the survey among the eight athletes who participated in the study are reported in Table 1. Results show that all athletes conducted self-administered training sessions with a slightly higher muscular strength training intensity of sessions with respect to aerobic/cardio ones. The overall duration of participants’ workouts allowed two separate clusters to be defined: athletes who trained for less than 45 min and, vice versa, the ones who trained more than 45 min. Finally, the survey indicated that the lockdown affected the training habits of athletes that continued with self-administered training sessions after the team activities resumed.

### 3.2. Instrumental Assessment: Distances Analysis

**Figure 2:**
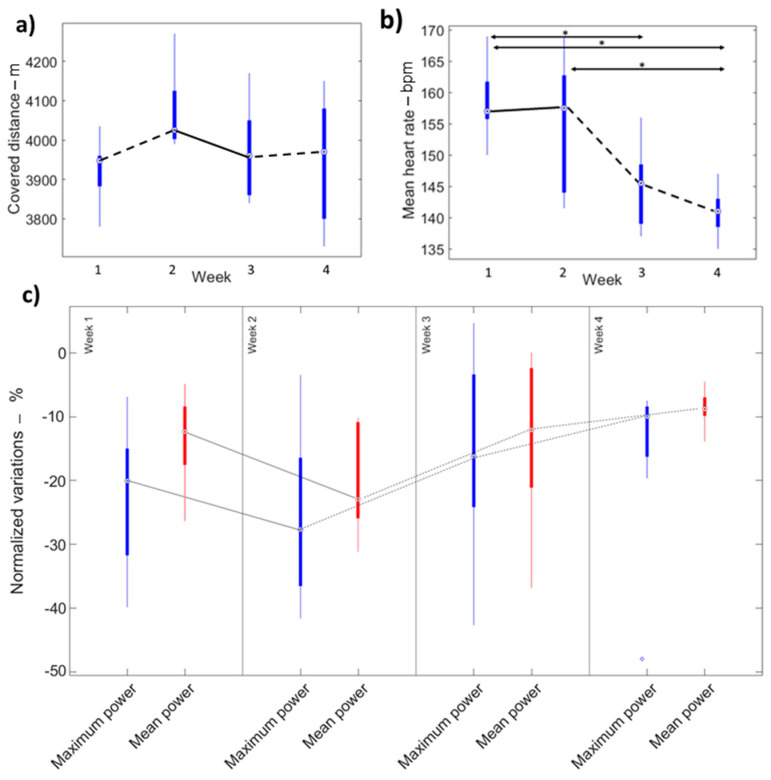
Boxplots and Bonferroni’s comparison for: (**a**) covered distance, (**b**) mean heart rate, (**c**) Wingate’s mean power and minimum power. Dashed lines indicate an incresead median performance, while solid lines indicate a decreased one.

**Figure 3:**
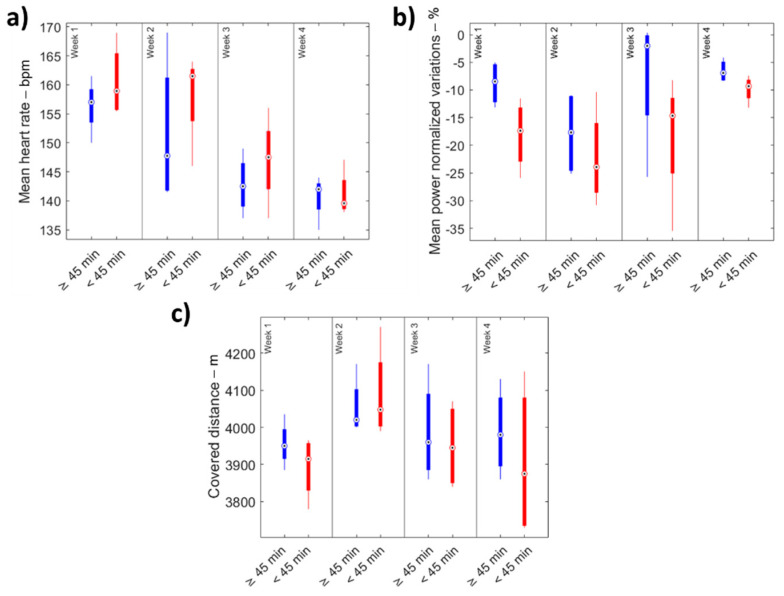
(**a**) Mean heart rate, (**b**) mean power normalized variations, and (**c**) total covered distance for the two different groups.

Friedman’s and Bonferroni tests did not show significant differences between the four weeks in terms of covered distances. (Figure 2). Low values of CQV confirmed negligible levels of inter-subject variability for covered distance (Table 2). No qualitative differences were found between athletes who trained for less than 45 min during the lockdown and the ones who trained more than 45 min (Figure 3).

### 3.3. Instrumental Assessment: Heart Rate Analysis

Concerning heart rate data, Friedman’s and Bonferroni tests showed significant differences between the first two and the last two weeks, thus suggesting a conceptual split in the data set in two main phases (Figure 2): in the first two weeks mean heart rate values did not significantly differ suggesting a maintained fatigue condition. Conversely, in the last two heart rate showed a decreasing trend along the weeks, suggesting athletes’ physiological adaptation. Low values of CQV confirmed negligible levels of inter-subject variability (Table 2).

Athletes who reported less than 45 min of daily training during the lockdown were characterized by higher mean heart rates (Table 3) and more significant variability between the four weeks (Figure 3) than the ones who trained for more than 45 min.

### 3.4. Instrumental Assessment: Wingate Tests

Results from the Wingate tests objectively confirm that fatigue reduces significantly the athletes’ ability to exert power. Indeed, a significant decrease in the exerted power was observed between the acquisitions taken before and after the fatigue protocol in all the four experiment weeks (i.e., Wilcoxon *p* < 0.05—Table 2).

As concerns the temporal evolution of the parameters normalized variations, Friedman’s test with Bonferroni post hoc analysis showed no significant differences between the four weeks (*p* > 0.05) for the mean and maximum power (Figure 2). Nevertheless, a two phases phase split (i.e., downfall—negative slope of the boxplots, and recovery—positive slope of the boxplots) is visible in the boxplots of both the mean and median power (Figure 2) and from Cliff’s delta effect sizes, which are characterized by the maximum value (i.e., the maximum effect of fatigue) in the second week and decreasing values in the third and fourth week (i.e., the minor effect of fatigue).

In addition, using questionnaire results to discriminate against athletes who trained for less than 45 min during the lockdown, we observed higher variations in the mean exerted power (Table 3 and Figure 3) than the ones who trained for more than 45 min.

### 3.5. Instrumental Assessment: Bosco Test

**Figure 4:**
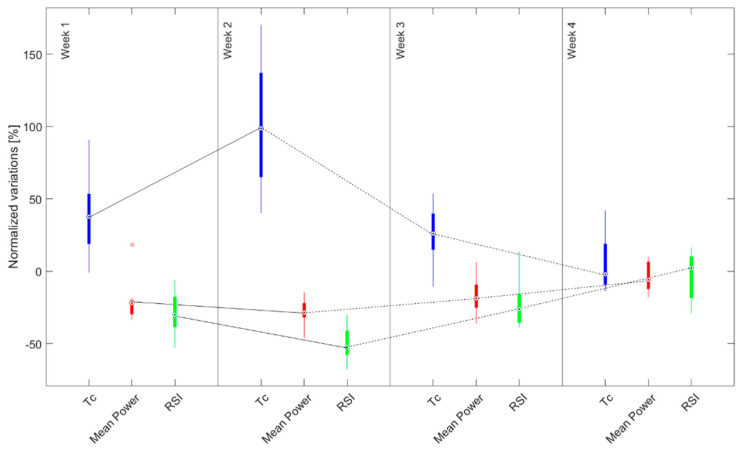
Boxplots of the parameters that were significantly influenced by the fatiguing protocol in the Bosco test. Dashed lines indicate an increased median performance, while solid lines indicate a decreased one.

**Figure 5:**
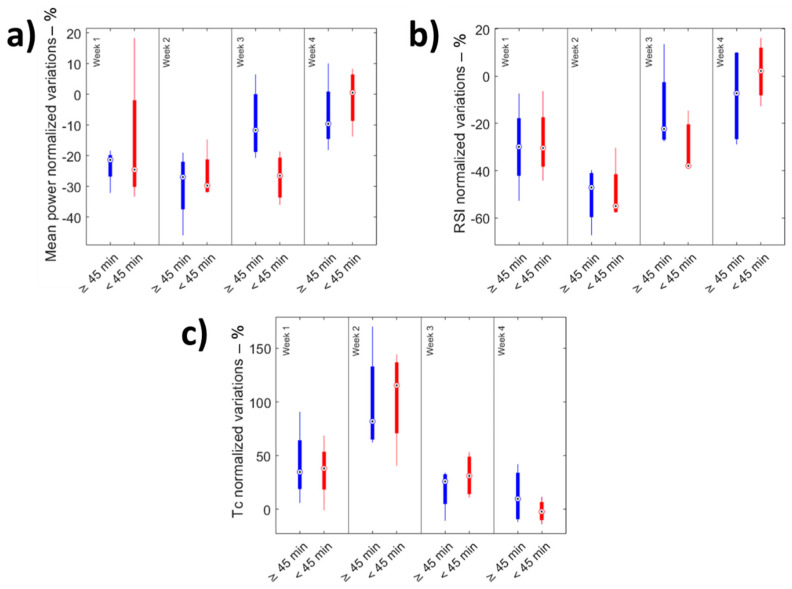
**(a**) Mean power, (**b**) RSI, and (**c**) *T_c_* normalized variations for the two different groups.

The Wilcoxon signed-rank test showed that three out of six parameters extracted from this test (i.e., mean power, RSI, *T_c_*) were significantly influenced by fatigue (*p* < 0.05—Table 4). The temporal evolution of these three parameters confirmed the behavior introduced by the Wingate test results (i.e., an initial downfall of the performance followed by a recovery—Figure 4). As for the mean heart rate, we could distinguish between two main phases: the first two weeks characterized by what appears to be a fatigue condition (i.e., low performances for all of the three significant parameters that become worse in the second week) and the last two weeks, in which an improvement of the parameters could be observed (i.e., the athletes can better adapt to the training protocol from a ballistic point of view). Friedman’s test resulted in significant differences (*p* < 0.05) for all the parameters. Figure 5 shows the comparison of the significant parameters between the groups that trained less and more than 45 min.

## 4. Discussion

This work started with the assumption that an extremely long forced-rest period and the self-isolation caused by the recent COVID-19 pandemic could have temporarily compromised the athletes’ muscular strength and performance [9]. Providing an instrumental analysis of football athletes at activities resume, this study offers a unique analysis of the biomechanical and physiological adaptation of a small (i.e., only eight participants) but homogenous (same team training before the pandemic) cohort of semi-professional football players after the lockdown period. In the light of the presented results, it can be confirmed that the two-month forced rest period caused a temporary weakening of the participants’ muscle mass. This hypothesis is further confirmed by the study recently proposed by Grazioli et al. [20], which shows how the lockdown impaired several physical performance measures compared with regular off-season. In this framework, it is clear that the forced rest period we experienced during the lockdown was extremely different from a normal off-season scenario. Indeed, it involved both physiological and psychological aspects resulting in a significant reduction in performance and physical ability. The degradation of performances after the lockdown could be explained by considering the fact that, even if athletes underwent several self-imposed training sessions, the working load was mainly unspecific, unsupervised, and at low intensities, resulting in the inability to correctly adhere to soccer-specific exercises. Indeed, the results of the qualitative questionnaire confirm these aspects on our pool of athletes, similar to what was previously observed on South African football players [7]. It is also worth noticing that most athletes continued to individually train after the lockdown, in addition to the team workouts. This also occurred for those athletes who were not used to carry out individual training sessions before the lockdown. Such positive change in habits is not observed here for the first time and confirms recent studies on the post-COVID phase [7]. Unfortunately, we do not have access to instrumental data acquired before the lockdown for these athletes that would be needed for a proper analysis of the time needed for a full recovery. However, experimental results showed a coherent behavior in the analyzed parameters: an initial downfall of the performance (with a negative peak in the second week), followed by a recovery in the last two weeks. It means that during the first days, the effect of fatigue was more evident than the other days, suggesting a physiological (heart rate) and biomechanical (Wingate and Bosco tests) adaptation of the participants.

Concerning the fatiguing protocol choice, we intended to reliably recreate the workload of a typical football match, which is characterized by frequent changes in direction and rapid explosive efforts, interrupted by short rest periods. The reproducibility of this method is suggested by results of the Friedman test with Bonferroni’s post hoc analysis on GNSS data, which found no significant differences in the total covered distance across the weekly training sessions. This standardized exercise allowed us to objectively measure the evolution of the athletes’ responses to the same effort. In addition, two kinds of instrumental responses were evaluated during training sessions in a one month period: (i) biomechanical performance by means of the Wingate and Bosco tests, which gave information on the athlete exerted power and ballistic ability respectively and (ii) physiological adaptation by means of the mean heart rate analysis during the training session. The reason for selecting the Wingate test, already adopted in several fields (i.e., exercise physiology, and physical medicine, and rehabilitation [16]) is that it allows for the monitoring of the lower-limb power exerted by the athlete (i.e., peak power, relative peak power, and mean power [17]). This aspect makes this test particularly suitable to evaluate athletic performance in football, in which the explosive ability of the athlete keeps a key role [18]. As concerns the Bosco test, it was used to evaluate the evolution of the athletes’ ballistic skills (i.e., vertical jump analysis) and it covered a different aspect of football (i.e., jumps and elevations). In our opinion these two complementary tests could give useful information about the participants’ performances in a hypothetical football match and, so, they allow us to completely monitor the athletes’ recovery. From a different viewpoint, heart rate monitoring provides physiological information about the athlete’s status and physical effort [19]. In particular, the increase in the heart rate depends on the intensity of the load [20] or, in other terms, the fatigued athletes’ are characterized by higher values of heart rate than the non-fatigued ones [21] because of the augmented request for oxygen. Indeed, it reflects the circulatory load and the intensity of energy metabolism in an indirect way [22]. Therefore, workload, heart rate, and oxygen uptake seem to be characterized by a linear relationship [22]. In this framework, heart rate monitoring during the execution of the same exercise at different times (e.g., different days) may provide useful information on the athlete’s aerobic recovery (e.g., as a response to a training/rehabilitation process).

Relevant differences between the first and last week (*p* < 0.05 for the mean heart rate) were found in accordance with studies on the fitness recovery in normal pre-season sessions [6,14,15], resulting in an instrumental confirmation of the improvement of the athletes’ fitness level. The need for recovery after the lockdown on heart rate can be further analyzed by observing its weekly evolution. The monitoring of the heart rate allowed us to distinguish between two main phases, the first one characterized by a not significant change in high values of mean heart rate values, while in the second phase a significant decrease in the mean heart rate could be observed (Friedman’s test and Bonferroni post-hoc analysis). Indeed, the first two weeks after activities resume are characterized by higher values for the same exercise, revealing lower physical adaptability and a higher sensitivity to fatigue with respect to the third and fourth week. A similar trend was further proved by the biomechanical analysis of the Wingate test, which reflects the athlete’s muscular strength. Indeed, the temporal analysis of the exerted mean and maximum power (i.e., boxplots of the normalized variations and Cliff’s delta effect size), showed the following pattern: initial decrease in the performance, with a minimum in the second week, followed by a recovery phase. Similar to the mean heart rate, this trend allowed us to distinguish two phases (i.e., fatigue in the first two weeks and recovery of the performance in the last two). The main difference with the heart rate lies in the second week. In particular, the physiological parameter maintained higher but no different values in the first two weeks. On the other hand, Wingate test parameters showed a deterioration of the values from the first to the second week, with the latter characterized by the worst performance. It is reasonable to assume that athletes’ explosive ability, results in more sensitivity to fatigue (i.e., the second week is worse than the first due to fatigue build-up) than to physiological changes (i.e., the mean heart rate is not statistical different between the two weeks). Nevertheless, Friedman’s test and Bonferroni’s least significant difference showed *p* > 0.05 for the Wingate parameters (i.e., mean and maximum power) across the weeks. This phenomenon could be explained by the fact that the pool of participants is limited to only eight athletes, limiting the power of a statistical analysis across the weeks. The aforementioned trend is also confirmed by the Bosco test outputs (i.e., mean power, RSI, *T_c_*), which were measured using single wearable inertial sensors, placed on the right foot. In addition, the Wingate test showed significant effects for all four weeks, comparing measures before/after effort, while for the Bosco test parameters differed only for the first three weeks. In other words, the performances before and after the fatigue protocol in the Bosco test suggested the recovery of the athletes’ ballistic ability was achieved at week four. These results could lead to two possible interpretations: (i) the Wingate test, results in more sensitivity to the fatiguing protocol than the Bosco test; and/or (ii) athletes recovered their ballistic ability faster (i.e., performance in the vertical jump) than their explosive ability (i.e., exerted power in the Wingate test).

In the lights of these results, it could be stated that athletes underwent an initial decrease in performance (i.e., see the boxplots of the normalized variations and Cliff’s delta effect sizes of the Wingate outcomes) followed by a recovery, which has a triple aspect: (i) a physiological one (i.e., reduction in the mean heart rate); (ii) an explosive one (i.e., Wingate test), which is characterized by a slow recovery (pre–post fatigue parameters keeps their significant difference after four weeks); and (iii) a ballistic one (i.e., the Bosco test), characterized by a recovery that is no longer detectable at week four. It could be speculated that in the downfall phase athletes were not able to metabolize the working load of the training sessions because of the muscle weakness due to prolonged rest periods. Elements to confirm this hypothesis comes from the analysis of two subgroups of athletes building on the amount of time spent training during the lockdown. Athletes’ who trained more than 45 min were characterized by lower and less variable mean heart rate values and smaller variations in the exerted power, showing better adaptability. On the other hand, participants’ who trained less than 45 min were characterized by higher heart rate values (e.g., minor aerobic ability), slower physical adaptation (e.g., higher heart rate variability), and bigger susceptibility to fatigue (e.g., higher power variations). Similar results were found for the Bosco test parameters analysis.

To the authors’ knowledge, no previous studies have monitored the recovery of the physiological behavior and ballistic/explosive performance after a prolonged and abnormal forced rest period like the one we have experienced last year. In particular, most of the research has focused on the comparison between the off-season and the end of the pre-season periods, or between the end of the off-season and the in-season, showing the negative effects of a long rest period and the positive outcomes of a training program after it. Only Grazioli et al. [20] analyzed the effects of the lockdown on the general fitness of twenty-three Brazilians soccer players. In particular, they compared the performances before and after the Brazilian forced rest period, finding that the lockdown was more detrimental than traditional off-season. However, as mentioned before, our work differs from the one proposed by Grazioli et al. Indeed, it focused on the recovery of the athletes’ physiological, ballistic, and explosive abilities in the month immediately after the lockdown, with the specific aim of better understanding the time required for a complete recovery of the performances. In this framework, the present work gives some preliminary information on the recovery trend of eight semi-professional athletes, providing a weekly analysis of the athletes’ physiological and biomechanical adaptation. Previous studies have shown that prolonged periods of detraining can induce a reduction in aerobic fitness, anaerobic power, and sprint ability [6]. Several studies showed improvement in aerobic fitness during the pre-seasonal period (i.e., the period immediately after the off-season one) due to the resumption of aerobic training [28,29]. Similar results were presented in our study. Indeed, the mean heart beat rate, which is an indirect measure of the aerobic consumption, was characterized by a weekly improved adaptation during the month immediately after the COVID-19 lockdown (i.e., lower values for the same exercise). As for the studies mentioned above, this increase in aerobic performance could be reasonably related to the return to training.

Caldwell at al. [6] found significant improvements in athletes’ pre-season anaerobic performance compared to the off-season due to basic and functional muscle strength training. The measurements were done before the start of the pre-season (July) training and at the end of it (August). Thus, the authors [6] did not analyze in detail the temporal evolution of the anaerobic ability during the pre-season period, but if we compare the Bosco parameters we analyzed in the proposed study during the very first and last weeks (i.e., similar to the analysis Caldwell et al. did in his study) we can confirm their results. Unfortunately, this difference was not statistically significant in our data, reasonably because of the limited number of participants; however, as mentioned in the Results section, the recovery trend is evident.

In the Silva et al. [9] review paper, it was found that after eight weeks, no pre- to post-preseason aerobic improvements were observed in endurance exercises. These results seem to be in contrast with the present study, in which the recovery of the physiological behavior and the ballistic performance was supposed to occur after four weeks (i.e., no significant differences in the mean heart rate between the third and fourth week and negligible effects of fatigue in Bosco test parameters). This phenomenon could be explained by considering that the participants of the present study did power (Wingate) and explosive tests (Bosco test) mainly, while their endurance ability was not tested. The main justification behind this choice is that we decided to test athletes’ soccer-specific abilities, which basically involve the capacity to exert a high amount of power in short periods.

Fessi et al. [30] measured significant improvements in vertical jump performances among seventeen athletes at activity resume after a classic off-season period. Unfortunately, a direct comparison is not appropriate, because in our study, the performance assessment before the activities interruption is unavailable. However, Fessi et al.’s observations of performance recovery, in five weeks, are in line with our study, in which the Bosco test parameters were observed to increase within the observation window at activities resume.

This study is not without limitations. The first open issue of this work is the lack of a pre-lockdown assessment and the lack of an accurate reporting of physical activities conducted during the lockdown. In particular, the proposed questionnaire was intended as a qualitative report and did not allow us to accurately estimate the amount of training.

Another limitation of the proposed study is the debated reliability of the Apple watch in estimating the heart rate at high intensities workouts. The scientific literature is not clear about the topic. Some studies [23,31] showed that the precision of the estimations decreases with the exercise speed, while others seemed to find good accuracies even during running [32,33]. As we found in the literature, the main problem is that the measurement rate could be not consistent especially at high intensities [23,31] but we can speculate that the missing heart rate values from the Apple Watch could be the ones evaluated physiologically implausible by the software and, so, discarded in the estimation of the mean heart rate [23]. Nevertheless, it is worth mentioning that the Apple watch resulted in being the most reliable device among several others for heart rate monitoring [32,33].

## 5. Conclusions

In conclusion, even if the pool of tested athletes was limited (n = 8), thanks to the instrumental approach we achieved some preliminary responses to better understand how such an unpredictable stop from team training would affect the response to fatigue of soccer players. Athletes’ biomechanical response was characterized by an initial rapid decrease in the performance (i.e., Wingate exerted power and Bosco test outputs) in the first two weeks with a negative peak in the second week, followed by an improvement phase due to recovery. In addition, physiological behavior (i.e., mean heart beat rate) followed a similar pattern characterized by a fatigue phase (i.e., first two weeks) and a recovery one (i.e., last two weeks). Instrumental data between the last two weeks did not differ significantly in the Bosco test, suggesting that a ballistic recovery would need at least 3–4 weeks of training. Elements indicative of a full recovery of explosive skills were not confirmed in the four weeks observation window.

## Figures and Tables

**Table 1:** The questionnaire administered to the athletes participating in the study.

**Did you carry out self-administred training sessions during the lockdown?**	
Yes	8 (100%)
No	0 (0%)
**Did you carry out aerobic/cardio exercises?**	
Yes	7 (88%)
No	1 (22%)
** How ofter did you do aerobic/cardio training in a week?**	
Daily	0 (0%)
Every alternate day	3 (43%)
2 times a week	4 (57%)
Once a week	0 (0%)
** How intense was your aerobic/cardio training on a scale from 1 to 10?**	6.35 ± 1.25
**Did you carry out muscular strength training?**	
Yes	6 (75%)
No	2 (25%)
** How often did you do strength training exercises in a week?**	
Daily	1 (16%)
Every alternate day	2 (33%)
2 times a week	2 (33%)
Once a week	1 (16%)
** How intense was your strength training on a scale from 1 to 10?**	6.75 ± 0.89
**During the lockdown, what was the overall duration of your workouts?**	
<30 min	1 (13%)
30–45 min	3 (37%)
45–60 min	3 (37%)
>60 min	1 (13%)
**Before COVID-19 lockdwon, were you used to perform any training activity in addition to the team training?**	
Several times a week	0 (0%)
Once a week	0 (0%)
Rarely	4 (50%)
No	4 (50%)
**After the COVID-19 lockdown, have you continued to perform additional self-administred training sessions?**	
Several times a week	3 (37%)
Once a week	2 (25%)
Rarely	2 (25%)
No	1 (13%)

**Table 2:** Median values, IQR and CQV for the covered distance, mean heart rate, mean and minimum power together eith the wilcoxon *p*-values and cliff’s delta effect size.

Covered Distance	Week 1	Week 2	Week 3	Week 4
**Distance, m**				
Median (IQR)	3948 (78)	4025 (123)	3960 (190)	3970 (280)
CQV	0.99	1.51	2.40	3.55
**Mean Heart Rate**				
**HR, bpm**				
Median (IQR)	157 (6)	158 (19)	146 (10)	141 (5)
CQV	1.89	6.11	3.30	1.59
**Wingate Test**				
**Mean Power**				
Pre-effort, median (IQR)	427 (118)	406 (118)	381 (147)	472 (71)
Post-effort, median (IQR)	394 (117)	334 (79)	332 (77)	442 (74)
*p*-value	<0.01	<0.05	<0.01	<0.01
Cliff’s Delta ES	0.48	0.69	0.50	0.35
**Maximum Power**				
Pre-effort, median (IQR)	515 (181)	483 (179)	468 (210)	600 (117)
Post-effort, median (IQR)	450 (195)	398 (63)	406 (146)	442 (74)
*p*-value	<0.01	<0.05	<0.01	<0.01
Cliff’s Delta ES	0.50	0.78	0.47	0.34

IQR = Interquartile Range, CQV = Coefficient of Quartile Variations, bpm = beats per minute.

**Table 3:** Median values, IQR of the heart rante and mean power normalized variations for each week in the two groups.

Mean Heart Rate, bpm	Week 1	Week 2	Week 3	Week 4
**≥45 min:** Median (IQR)	157 (6)	148 (20)	143 (8)	142 (5)
**<45 min:** Median (IQR)	159 (10)	162 (9)	148 (10)	140 (5)
**Mean Power Normalized Variations, %**				
**≥45 min:** Median (IQR)	−8.5 (6.9)	−17.7 (13.5)	−2.0 (14.5)	−6.9 (3.4)
**<45 min:** Median (IQR)	−17.4 (9.8)	−23.9 (12.6)	−14.7 (13.6)	−9.3 (3.4)

**Table 4:** Wilcoxon test *p*-value (Cliff’s delta) shadowed cells indicates a significant effect of the fatiguing protocol on Bosco test parameters.

Parameters	Week 1	Week 2	Week 3	Week 4
*h_max_*	>0.05	**<0.05**	>0.05	>0.05
Mean power	**<0.05**	**<0.05**	**<0.05**	>0.05
RSI	**<0.05**	**<0.05**	**<0.05**	>0.05
Stiffness	>0.05	**<0.05**	**<0.05**	>0.05
*T_c_*	**<0.05**	**<0.05**	**<0.05**	>0.05
*T_f_*	>0.05	**<0.05**	>0.05	>0.05

## Data Availability

Participants data, the full dataset and statistical code are available from the corresponding author (luigi.truppa@santannapisa.it). Participant consent for data sharing was not obtained but the presented data are anonymised and risk of identification is extremely low.
